# Performance comparison of three *BRAF* V600E detection methods in malignant melanoma and colorectal cancer specimens

**DOI:** 10.1007/s13277-014-2711-5

**Published:** 2014-10-16

**Authors:** Inger Marie Løes, Heike Immervoll, Jon-Helge Angelsen, Arild Horn, Jürgen Geisler, Christian Busch, Per Eystein Lønning, Stian Knappskog

**Affiliations:** 1 Department of Clinical Science, University of Bergen, Post box 7804, 5021 Bergen, Norway; 2 Department of Oncology, Haukeland University Hospital, Bergen, Norway; 3 Department of Pathology, Ålesund Hospital, Ålesund, Norway; 4 Department of Clinical Medicine, University of Bergen, Bergen, Norway; 5 Department of Digestive Surgery, Haukeland University Hospital, Bergen, Norway; 6 Institute of Clinical Medicine, University of Oslo, Oslo, Norway; 7 Present Address: Department of Oncology, Akershus University Hospital, Lørenskog, Norway; 8 Present Address: Aleris Helse, Marken 34, 5017 Bergen, Norway

**Keywords:** *BRAF* mutations, Detection methods, Immunohistochemistry, Colorectal cancer, Malignant melanoma

## Abstract

Personalized cancer care requires reliable biomarkers. While the *BRAF* V600E mutation is implemented in the clinic, no method for its detection has so far been established as reference. We aimed to perform a comprehensive comparison of three methods currently being used for V600E detection in clinical samples. We analysed genomic DNA from 127 malignant melanomas (77 patients) and 389 tumours from 141 colorectal cancer patients (383 liver metastases and 6 primary tumours) by Sanger sequencing and a single probe-based high-resolution melting assay (LightMix). Formalin-fixed paraffin-embedded (FFPE) tissue from a subset of these lesions (*n* = 77 and 304, respectively) was analysed by immunohistochemistry (IHC) using the V600E-specific antibody VE1. In a dilution series of V600E-mutated DNA in wild-type DNA, the detection limit for the LightMix assay was 1:1000 mutated alleles while it was 1:10 for Sanger sequencing. In line with this, we detected 15 additional mutated melanoma samples and two additional mutated metastatic colorectal cancer samples by the LightMix assay compared to Sanger sequencing. For the melanoma samples, we observed high concordance between DNA-based methods and analysis by IHC. However, in colorectal samples, IHC performed poorly with 12 samples being scored as V600E positive exclusively by IHC and nine samples being scored as V600E negative exclusively by IHC. In conclusion, the VE1 antibody is not recommendable for clinical tests of colorectal cancer samples. For melanoma samples, IHC may be useful as a screening tool guiding further analytical approaches.

**Electronic supplementary material:**

The online version of this article (doi:10.1007/s13277-014-2711-5) contains supplementary material, which is available to authorized users.

## Introduction

In the era of personalized cancer care, reliable detection methods for biomarkers are utterly important. A biomarker already implemented in the clinic is the *BRAF* V600E mutation. In malignant melanoma (MM), this mutation (and to a smaller extent the less frequent V600K mutation) indicates utility of *BRAF* inhibitors (such as vemurafenib and dabrafenib) [1, 2], whereas in colorectal cancer (CRC), V600E is suspected to account for resistance to EGFR antibodies in patients harbouring *KRAS* wild-type tumours [3, 4] as well as indicating a dismal prognosis [5]. Currently, the *BRAF* V600E is also evaluated in CRC in algorithms for screening for Lynch syndrome [6, 7].

For several years, Sanger sequencing has been considered the reference method for detection of specific mutations in human tumours, including *BRAF* V600E and V600K [8], although a “gold standard” detection method for these mutations in diagnostic laboratories is yet to be established. Undoubtedly, with the growing interest in biomarkers, massive parallel sequencing will soon become cost-effective and will, in the coming years, be implemented in diagnostic pathology laboratories. This technology will provide *BRAF* status along with a large number of other biomarkers. However, single biomarker assays will still remain important as confirmative assays, and they will also be used for cito diagnostics and in low-quality tissues. Regarding targeted analysis of *BRAF* V600E, Capper et al. reported in 2011 a novel mutation-specific antibody (VE1) allowing detection of V600E-mutated cells by immunohistochemistry (IHC) in paraffin-embedded archive samples (FFPE) [9]. The sensitivity as well as specificity for this antibody was reported to be 100 % for both malignant melanoma species and in papillary thyroid carcinoma. While some subsequent studies have confirmed the value of the VE1 antibody in several tumour forms [10–16], notably, it has also been reported to be of uncertain value in analyses of colorectal carcinomas, due to insufficient sensitivity [17].

For detection of the *BRAF* V600E mutation at the DNA level, several methods including Sanger sequencing, pyro-sequencing, high-resolution melting assays (HRMAs), dHPLC, TaqMan assays as well as massive parallel sequencing have been utilized [18–23]. Notably, in the BRIM-3 phase 3 trial, leading to vemurafenib being approved for clinical use in patients with metastatic MM, a real-time PCR assay (cobas 4800 *BRAF* V600 Mutation Test, Roche Molecular Systems) was used to define mutation status [1].

Some investigators have suggested that IHC might be used as a first-line method to screen for the V600E mutation due to its high specificity, while DNA-based methods should be performed for samples scored as staining-negative or uninterpretable cases [8, 23]. This approach resembles to a large extent the strategy established for HER2 testing in breast cancer, where IHC is used for initial testing, while CISH/FISH is applied for cases scored as 2+ by IHC staining (Norwegian national guidelines; www.nbcg.no, April 2014).

Here, we present a comprehensive comparison of three *BRAF* V600E detection methods including immunohistochemistry (IHC) using the mutation-specific monoclonal antibody (VE1), Sanger sequencing and a single probe-based high-resolution melting assay (LightMix) with a clamped wild-type allele amplification, leading to an expected higher sensitivity. Importantly, we compare all three methods across both melanoma and colorectal cancer specimens.

## Material and methods

### Patient specimens

**Fig. 1:**
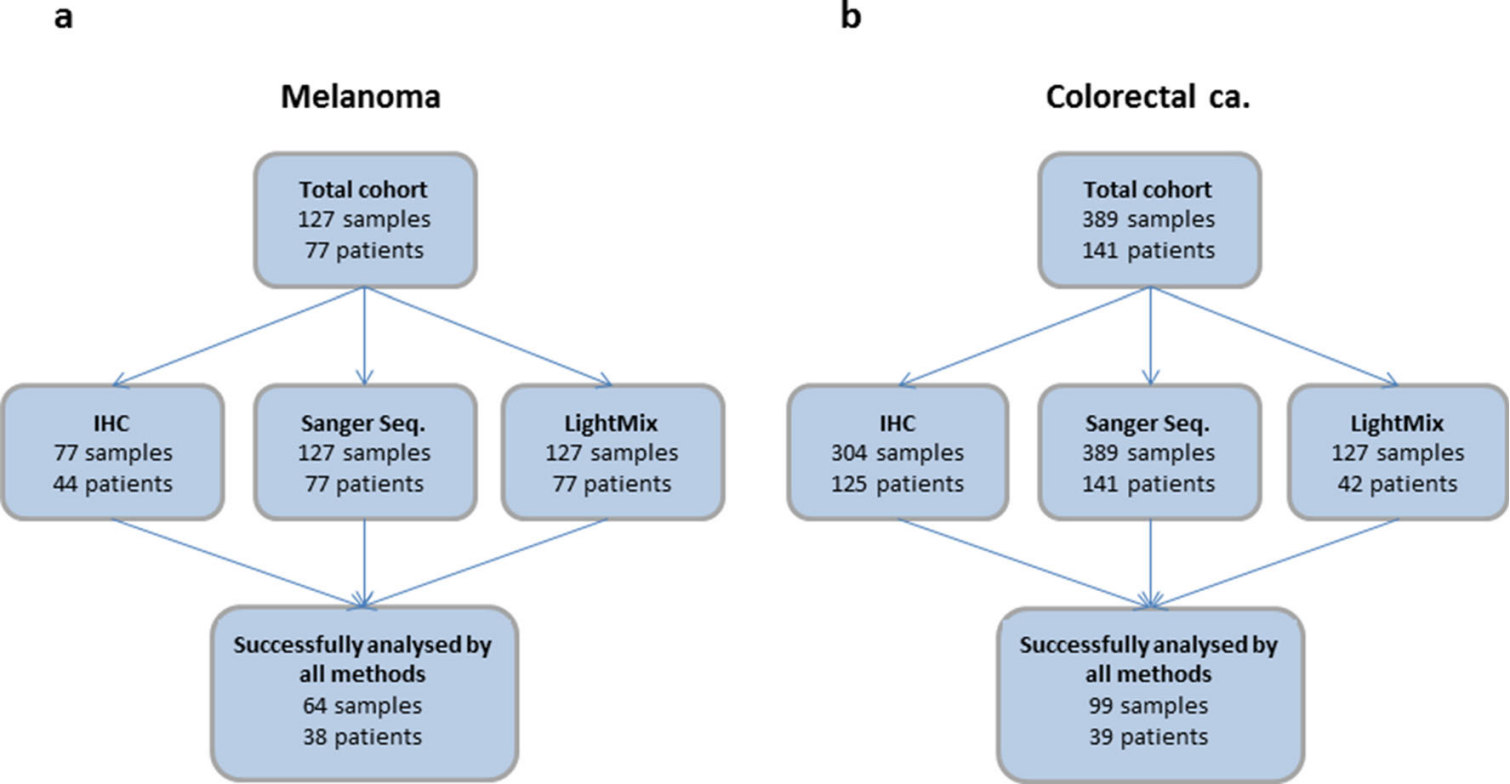
Flow chart illustrating the number of samples and patients analysed by the three different methods (**a**; melanoma, **b**; colorectal cancer). The subset of samples analysed by the LightMix assay for the CRC samples includes both positive and negative samples as characterized by Sanger sequencing as well as all discrepant cases within samples from one patient and between methods (Sanger vs IHC). For direct comparison between methods, we used the 64 melanoma samples and the 99 colorectal samples successfully analysed by all three methods. Notably, two of the melanoma samples were not interpretable by the LightMix assay. Further, 13 of the melanoma samples and 19 of the CRC samples were not interpretable by IHC (for details, see Supplementary Table S1)

The numbers of samples analysed with the three different methods are listed in Fig. 1. Genomic DNA (gDNA) from 127 metastatic deposits obtained from 77 patients with metastatic malignant melanoma was selected from a previously described study including a total of 85 patients. *BRAF* V600 status assessed by Sanger sequencing has previously been reported for these patients [24]. Here, we did not analyse samples previously found to be *NRAS* mutated, as *BRAF* and *NRAS* mutations are mutually exclusive [25, 26]. Formalin-fixed paraffin-embedded (FFPE) tissue from 77 metastases from 44 of these patients was available for IHC staining.

Genomic DNA was isolated from 389 tumours from 141 patients suffering from metastatic colorectal cancer (mCRC) (383 surgically removed liver metastases and six primary tumours). Further, FFPE tissue from 304 liver metastases from 125 of these patients was subjected to immunohistochemical staining. Thus, for most patients, two or more individual lesions were analysed.

Genomic DNA from all patient samples used was extracted from fresh frozen tissue using the QIAamp DNA Mini Kit, (QIAGEN, Hilden, Germany) according to the manufacturer’s instructions, and the two DNA-based methods were performed on DNA from the same extracted aliquot for each sample.

One part of each biopsy sampled was, if possible, formalin-fixed and paraffin-embedded for assessment of tumour content. For direct comparison between methods, we used the 64 melanoma samples (from 38 patients) and the 99 colorectal samples (from 39 patients) successfully analysed by all three methods (Fig. 1).

The sample collection was approved by the regional ethical committee, and all patients provided written informed consent before tissue sampling.

### DNA pre-amplification

Genomic DNA (gDNA) from the metastatic colorectal cancer samples was globally amplified using the REPLI-g Midi Kit (QIAGEN, Hilden, Germany) according to the manufacturer’s instructions. In brief, gDNA was incubated in denaturation buffer for 3 min before addition of neutralization buffer and a master mix containing phi29 DNA polymerase with exonuclease proofreading activity. The isothermal amplification reaction was carried out overnight at 30 °C. This procedure was performed in order to save patient material in our research laboratory; however it is not required to perform the analyses evaluated.

### Dilution series

In order to generate a dilution series for assessment of sensitivity limits for the DNA-based methods, we used PCR products carrying wild-type *BRAF* or *BRAF* V600E. These PCR products were generated from a cell line (A2058) heterozygote for the V600E mutation. We added known amounts of mutated DNA fragments to the wild-type products. The ratio spanning from 1:1 to 1:10^7^ mutated vs wild-type molecules.

### PCR amplification and Sanger sequencing

*BRAF*, exon 15 (harbouring codon 600), was amplified using the DyNazyme EXT polymerase system (Finnzymes, Espoo, Finland) according to the manufacturer’s instructions with forward primer 5′-tca taa tgc ttg ctc tga tag ga-3′ and reverse primer 5′-ggc caa aaa ttt aat cag tgg a-3′. Thermocycling conditions were an initial step at 94 °C for 5 min, 35 cycles at 94 °C for 30 s, 56 °C for 30 s and 72 °C for 30 s, followed by a final step at 72 °C for 7 min. The resulting PCR products were sequenced using Big Dye v1.1 terminator mixture (Applied Biosystems, Carlsbad, CA). All sequencing reactions were carried out with the same primers as used for PCR amplification. For most samples, only the forward primer was used, but all alterations other than V600E were verified by a second sequencing reaction using the reverse primer. After an initial step of 5 min denaturation at 94 °C, the sequencing reaction was carried out for 30 cycles of 15 s at 94 °C, 5 s at 50 °C and 4 min at 60 °C. Capillary gel electrophoresis, data collection and sequence analyses were performed on an automated ABI 3700 DNA sequencer (Applied Biosystems, Carlsbad, CA). PCR amplification and Sanger sequencing of the CRC samples were performed on pre-amplified gDNA. All alterations detected were verified in an independent amplification using original gDNA as template.

### High-resolution melting assay

For high-resolution melting analysis of V600 status, we used a single probe-based assay specifically designed to detect both *BRAF* V600E and V600K (LightMix® Kit *BRAF* V600E/K, 2013 version, including a control reaction; TIB MOLBIOL, Berlin, Germany) according to the manufacturer’s instructions. Two separate reactions were performed for each sample: a mutation-specific reaction where amplification from *BRAF* wild-type template was clamped and a control reaction included in order to verify DNA quality in samples negative for the mutation-specific reaction. All reactions were performed using the Roche Diagnostics LigthCycler FastStart DNA Master Hybridization Probe reaction mix and run on a LigthCycler 480 instrument (Roche, Basel, Switzerland). The thermocycling conditions were as follows: denaturation at 95 °C for 10 min, amplification of target DNA; 25 cycles at 95 °C for 5 s, 58 °C for 10 s and 72 °C for 15 s; cycling and quantification through 40 cycles at 95 °C for 5 s, 81 °C for 30 s, 58 °C for 5 s acquisition, 58 °C for 10 s and 72 °C for 15 s; melting at 95 °C for 20 s, 58 °C for 20 s, 43 °C for 20 s and then a gradual increase in temperature to 75 °C with continuous acquisition before cooling at 40 °C. Genotype calling based on melting curves was performed using the LightCycler 480 software release 1.5.1 (Roche).

### Immunohistochemistry

Immunohistochemical staining was performed on 3–4 μm sections of FFPE tissue (the mCRC samples were arranged in tissue microarrays (TMAs), for the MM samples whole section slides were used), placed on coated glass slides and dried at 70 °C for 30 min. The staining procedure was performed the same day or the day after the sectioning on a Ventana BenchMark XT immunostainer (Ventana Medical Systems, Tucson, AZ). The sections were de-paraffinized using EZ Prep (Ventana Medical Systems) at 72 °C for 4 min. Heat pre-treatment for epitope retrieval was performed using CC1 solution (Ventana) containing Tris/borate/EDTA (pH 8.2) for 64 min at 99 °C. Slides were incubated with *BRAF* V600E-specific monoclonal mouse antibody clone VE1 (Spring Bioscience, Pleasanton, CA) diluted 1:60 in an antibody diluent (50 mM Tris, 150 mM NaCl, 1 % BSA, 0.01 % Na-Acid, 0.05 % Tween 20; pH 7.5), at 36 °C for 16 min. Following incubation with primary antibody, slides were treated with peroxidase inhibitor (3 % H_2_O_2_) for 4 min, HQ universal linker for 8 min, HRP multimer for 8 min, DAB for 8 min and copper (copper sulphate, 5 g/l) for 4 min, at 37 °C. Primary antibody detection was performed using the OptiView universal DAB Detection Kit from Ventana. Counterstaining was performed with Harris haematoxylin (Histolab Products AB, Gothenburg, Sweden) for 30 s at room temperature. Finally, the sections were blueing in running tap water for 2 min, dehydrated in alcohol solutions and xylene and mounted in Mountex (Histolab Products AB, Gothenburg, Sweden). A detailed description of all steps in the establishment of the IHC procedure is included as a separate section in the online supplementary information.

**Fig. 2:**
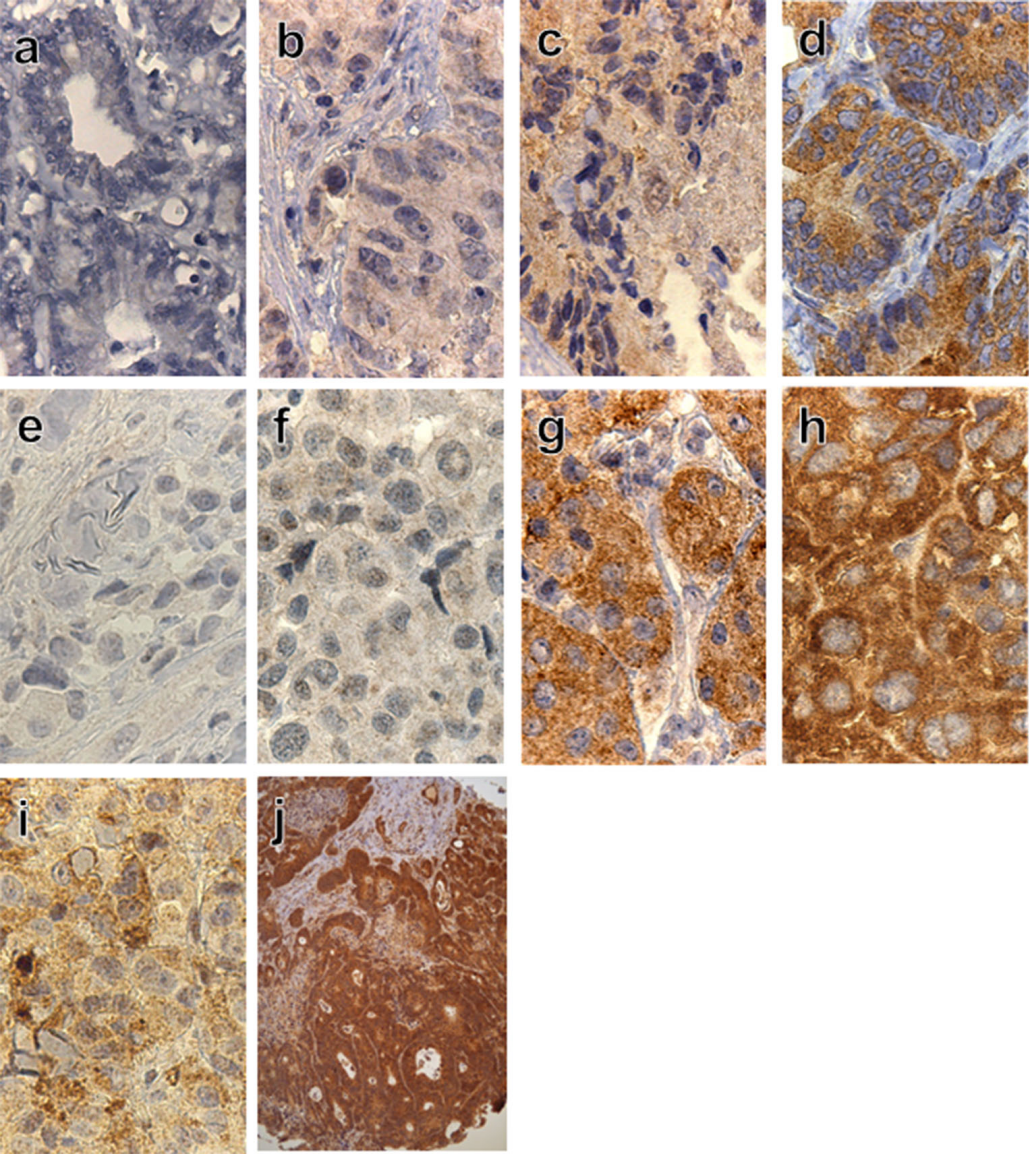
Representative examples of immunostaining distribution and intensity observed in metastatic colorectal cancer samples (**a**–**d**) and malignant melanoma samples (**e**–**h**). Grading from negative to grade 3+ for V600E (*left* to *right*). V600K mutated heavily pigmented MM sample (**i**). TMA section demonstrating the uniform staining throughout all tumour tissue (**j**). Original magnification: 400× (**a**–**i)**, 100× in (**j)**

*BRAF*-VE1 staining was seen exclusively in the cytoplasm. The intensity of immune staining was graded 0 if no visible staining, grade 1 if weak diffuse cytoplasmic background staining, grade 2 if moderate diffuse and granular cytoplasmic and grade 3 if strong mainly granular cytoplasmic staining (Fig. 2). No staining and staining grade 1 were regarded as negative for V600E. Grade 2 and grade 3 were regarded as positive, as described by Bösmüller and Sinicrope [10, 14]. In the positive samples, the staining was homogenous with equal intensity throughout the majority of tumour cells. Each slide was evaluated and scored independently by two of the authors (I. M. L. and H. I.) blinded to the V600E screening results from the two DNA-based methods. Any inconsistency was discussed and agreed upon. In any case of discordance showing positive results by IHC and negative mutation status by the DNA-based methods in the MM samples, a new slide from the same tumour was stained with haematoxylin only to detect and locate any pigmentation of the tumour. If the haematoxylin-stained slide revealed melanin (Fig. 2i) in all parts of the tumour, we would change our conclusion to negative staining.

### Evaluation of tumour content in biopsies

Before TMA production, all sections from the colorectal samples were haematoxylin and eosin (HE) stained and evaluated for tumour content, including viable tumour cells, necrosis and fibrosis as well as amount of normal tissue by an experienced pathologist (H. I.). The amount of viable tumour cells in the whole section slides was on average 81 % (range 10–100 %) with only five samples <30 %. The TMA cores were made from areas found to contain viable tumour cells, and then again evaluated by HE staining to ensure sufficient tumour content in each core. Each TMA core was 1 mm in diameter, and we used four cores from each sample to ensure representative material (no differences between the four cores from individual samples were recorded).

## Results

### Detection limits for DNA-based *BRAF* V600E analyses

**Fig. 3:**
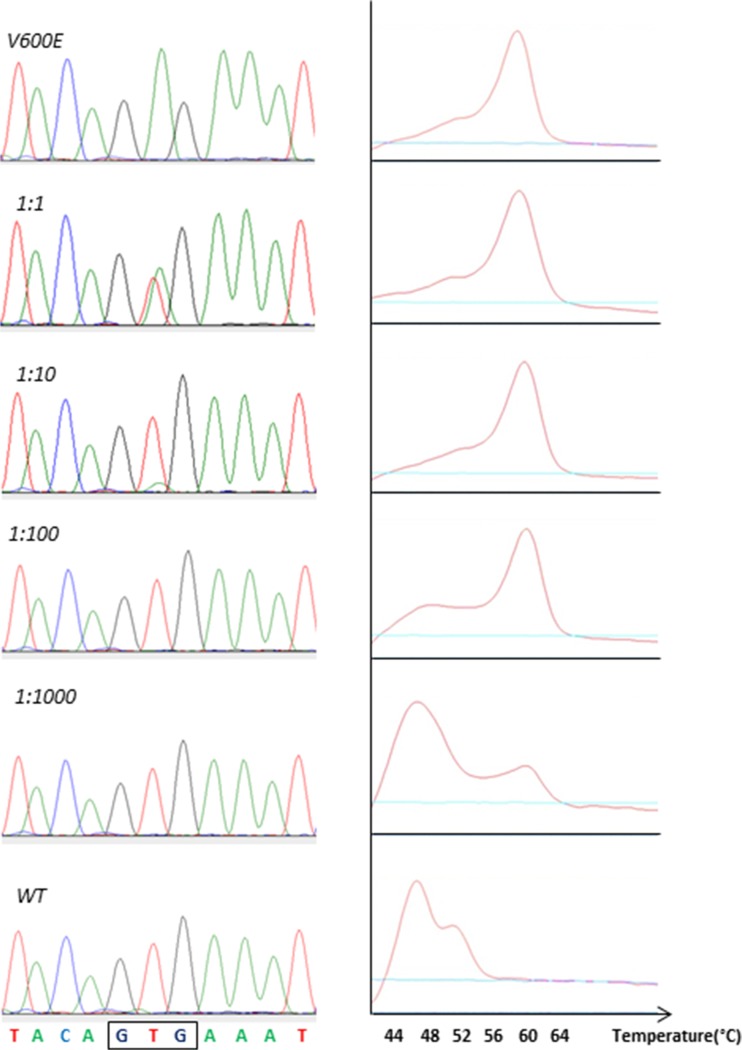
Electropherograms and melting profiles (from Sanger sequencing (*left*) and the LightMix assay (*right*)) for a dilution series containing a ratio of 1:1–1:10^7^ *BRAF* V600E-mutated DNA in *BRAF* wild-type DNA. (Only the four dilutions up to 1:10^3^ shown as all higher dilutions gave results resembling wild-type samples)

In order to establish the detection limits for the DNA-based methods used in the present comparison (Sanger sequencing and the LightMix high-resolution melting assay) with respect to detectable fraction V600E-mutated molecules among *BRAF* wild-type molecules, we performed analyses on a dilution series containing *BRAF* V600E mutated in *BRAF* wild-type DNA spanning a ratio from 1:1 to 1:10^7^. We found the detection limit for Sanger sequencing to be 1:10 while it was 1:1000 mutated alleles for the LightMix assay (Fig. 3).

### *BRAF* analyses in melanoma specimens

Detailed results for each individual sample are listed in Supplementary Table S1. Among 127 samples from 77 patients suffering from malignant melanoma, we found 60 samples from 31 patients to be positive for *BRAF* mutations (*n* = 28, V600E; *n* = 3, V600K) by Sanger sequencing. Analysing the same samples by LightMix high-resolution melting assay, we found 75 mutated samples from 37 patients to harbour *BRAF* V600 mutations. Thus, 15 samples classified as harbouring wild-type *BRAF* by Sanger sequencing were found to harbour V600 mutations (*n* = 14, V600E; *n* = 1, V600K) by the LightMix assay. In all of the 60 samples found mutation positive by Sanger sequencing, the same mutation was confirmed by the LightMix assay, including the V600K mutation.

**Fig. 4:**
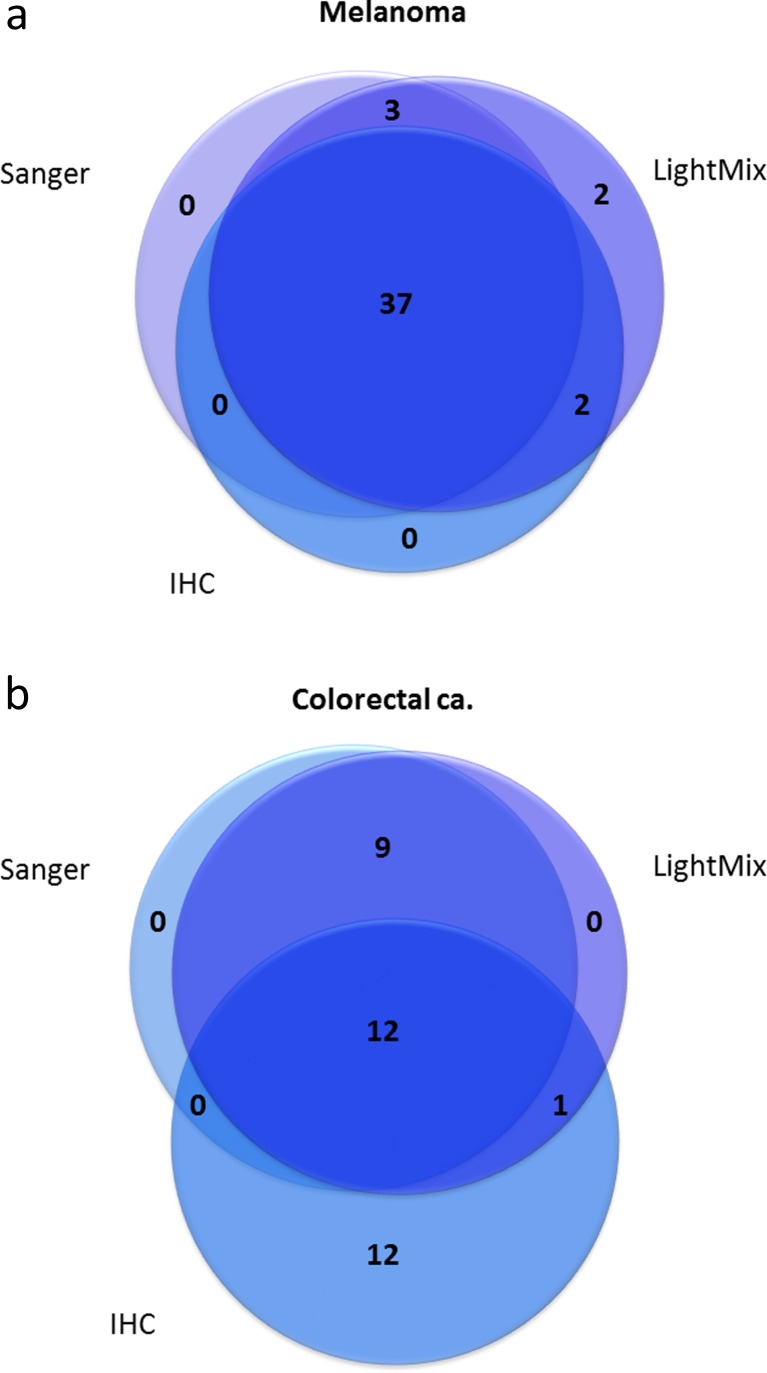
Venn diagrams illustrating the degree of concordance between the three *BRAF* V600E detection methods evaluated for malignant melanoma samples (**a**) and colorectal cancer samples (**b**). Only samples analysed by all three methods and yielding positive results in at least one analysis are included (melanoma: *n* = 44, colorectal cancer: *n* = 34)

Using the V600E-specific antibody VE1, we performed immunohistochemical analysis on 77 samples from 44 patients. We found 39 samples from 24 patients to be positive for V600E immunostaining. In total, 64 melanoma samples from 38 patients were successfully analysed by all three methods. A summary of the results for these 64 samples is listed in Table 1. Out of the 64 samples, 44 samples yielded positive results by at least one of the analytical methods (Fig. 4a). Seven samples yielded discordant results: Five samples for which the V600E mutation had been detected by the LightMix assay were scored as negative by IHC; out of these, three samples had been recorded as positive for V600E by Sanger sequencing. Further, two samples were scored as positive by the LightMix assay and IHC but negative by Sanger sequencing.

**Table 1:** *BRAF* V600E mutation screening results in malignant melanoma samples

Screening results			Number of samples	
wt by all methods			16	
V600E by all methods			37	
V600K by DNA methods			4	
Other exon 15 mutations			0	
Discordant^a^			7	
Total			64	
^a^Distribution of results in discordant samples				
Sample ID	Sanger	LightMix	IHC	Percentage of tumour cells
MM10-1	wt	V600E	V600E	50
MM25-3	V600E	V600E	wt	60
MM61-2	wt	V600E	V600E	20
MM61-3	V600E	V600E	wt	80
MM61-4	V600E	V600E	wt	70
MM71-2	wt	V600E	wt	<10
MM78-1	wt	V600E	wt	30

Four of the samples tested by IHC had been found to harbour the V600K mutation by the DNA-based assays. As expected, none of the tumours harbouring the V600K mutation stained positive, and these samples thus represent true negative controls.

### *BRAF* analyses in colorectal cancer specimens

Although *BRAF* V600E is most applicable as a biomarker in malignant melanoma, we aimed to extend our methods-comparison to colorectal cancer, where *BRAF* status may also have important implications.

Among 389 samples from 141 patients suffering from metastatic colorectal cancer (mCRC), we found 22 samples from seven different patients to be *BRAF* V600E mutated by Sanger sequencing. In a selected subset of these samples (*n* = 127), the LightMix assay identified the same mutations. In addition, two samples yielding negative results by Sanger sequencing were found to harbour the V600E mutation by the LightMix assay. The subset of samples analysed by the LightMix assay included both positive and negative samples as characterized by Sanger sequencing as well as all discrepant cases within samples from one patient and between methods (Sanger vs IHC).

Immunohistochemistry using the mutation-specific monoclonal antibody VE1 was performed on TMAs including tissue from 304 metastases from 125 of the mCRC patients. Twenty-five samples from 15 patients were found to be positive for V600E immunostaining. In total, 99 colorectal samples from 39 patients were successfully analysed by all three methods. A summary of the results for these 99 samples is listed in Table 2 (detailed results for each individual sample are listed in Supplementary Table S1). Out of the 99 samples, 34 samples yielded positive results by at least one of the analytical methods (Fig. 4b). Two samples revealed non-V600E and non-V600K mutations, and 22 samples yielded discordant results: Nine samples for which the V600E mutation had been detected by the LightMix assay were scored as negative by IHC; out of these, all nine had been recorded as positive for V600E by Sanger sequencing. Further, one sample was scored as positive by the LightMix assay and IHC but negative by Sanger sequencing, and 12 samples were scored as positive by IHC but negative by both DNA-based methods.

**Table 2:** *BRAF* V600E mutation screening results in colorectal cancer samples

Screening results			Number of samples	
wt by all methods			63	
V600E by all methods			12	
V600K by DNA methods			0	
Other exon 15 mutations			2	
Discordant^a^			22	
Total			99	
^a^Distribution of results in discordant samples				
Sample ID	Sanger	LightMix	IHC	Percentage of tumour cells
t9-1	wt	wt	V600E	100
t9-3	wt	wt	V600E	100
t14-1	wt	wt	V600E	100
t15-1	wt	V600E	V600E	80
t26-5	wt	wt	V600E	80
t29-4	wt	wt	V600E	100
t30-2	wt	wt	V600E	20
t39-1	V600E	V600E	wt	100
t39-2	V600E	V600E	wt	90
t39-3	V600E	V600E	wt	90
t49-2	wt	wt	V600E	40
t58-8	wt	wt	V600E	50
t64-3	wt	wt	V600E	80
t64-4	wt	wt	V600E	80
t67-2	wt	wt	V600E	20
t75-1	V600E	V600E	wt	100
t75-3	V600E	V600E	wt	100
t84B-1	V600E	V600E	wt	100
t99-2	V600E	V600E	wt	100
t99-3	V600E	V600E	wt	90
t99-4	V600E	V600E	wt	60
t156-1	wt	wt	V600E	100

### Non-V600E/V600K mutations

**Fig. 5:**
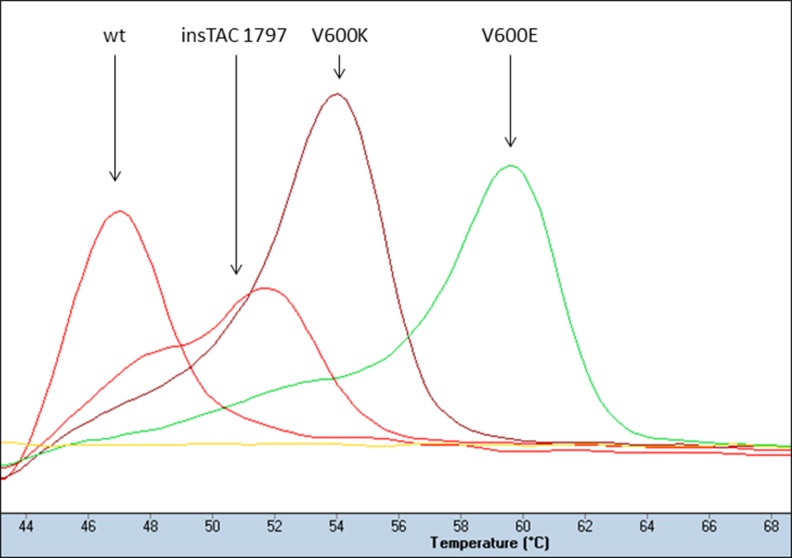
Melting curve profiles illustrating the detection of a non-V600E/V600K *BRAF* mutation (c.1796_1797insTAC) by a shifted melting temperature in the LightMix assay

Two non-V600E/V600K mutations were detected by Sanger sequencing: a substitution at *BRAF*-coding nucleotide 1780 (c.1780G > A; COSMIC ID: COSM27639) and a three-nucleotide insertion at coding nucleotide 1797 (c.1796_1797insTAC; COSMIC ID: COSM30730), previously found to cause hyperactivity of *BRAF* similar to V600E [27]. The G1780A mutation was not detected by the LightMix assay as it is designed to detect V600E and V600K mutations only. However, due to its close location to the V600, the c.1796_1797insTAC mutation was indicated in the melting point analyses as a shift in melting temperature (Fig. 5). Notably, none of these two mutations were detected by IHC using the VE1 antibody.

### Costs, time consumption and tissue requirements

Costs, time consumption and amount of material used for the three methods are listed in Table 3. Regarding costs, the two DNA-based methods would be comparable at approximately 5 USD/sample. IHC would cost approximately 20 USD per slide stained. However, using TMAs (in a research setting) would allow for low costs for IHC as multiple samples may be fitted on a single slide.

**Table 3:** Cost, time consumption and material input comparison of *BRAF* V600E detection methods

	Cost per sample (USD)	Time consumption (hands on)	Material used
Sanger sequencing^ab^	8	9 h (1 h)	50–100 ng
LightMix assay^ac^	4, 5	4 h (1 h)	100–250 ng
Immunohistochemistry^d^	20	4 h (15 min)	3–4 μm section

^a^Time and costs for DNA isolation not included

^b^Capillary gel electrophoresis, data collection and sequence analyses were performed on an automated DNA sequencer (ABI 3700)

^c^All reactions were run on LigthCycler 480 instrument (Roche), and genotype calling based on melting curves was performed using the LightCycler 480 software release 1.5.1 (Roche)

^d^The staining procedure was performed on a Ventana BenchMark XT immunostainer (Ventana Medical Systems)

The LightMix analysis is completed in less than 4 h while PCR and Sanger sequencing require at least 1 day. In both cases, DNA must be isolated up front. IHC will not require DNA isolation and thus is more readily available in a diagnostic laboratory, but evaluation of stained sections by microscopy is needed.

The input of gDNA used to run the DNA-based methods on patient samples was typically in the range of 50–100 ng for the Sanger sequencing and 100–250 ng for the LightMix assay respectively.

## Discussion

Personalized medicine introduces several new questions of importance to clinicians as well as researchers. As new biomarkers are identified and implemented in the clinic to guide treatment decisions, it is essential that reliable methods to detect these alterations are available. Regarding the *BRAF* V600E mutation, it has already been implemented as a predictive factor guiding therapy in malignant melanoma and is currently explored as a potential predictive marker in colorectal cancer as well as in other malignancies.

The V600E mutation occurs within the activating segment of the kinase domain and leads to constitutive activation of the mitogen-activated protein kinase (MAPK) pathway promoting cell proliferation and preventing apoptosis [28]. Later publications have pointed out the importance of recognizing other activating mutations in the same region [29]. The second most common *BRAF* V600 mutation (V600K) which has demonstrated increased kinase activity as well [30] is currently accepted as a predictive marker with respect to anti-*BRAF* therapy.

As massive parallel sequencing is continuously becoming cheaper and more efficient, this technology will undoubtedly be implemented in diagnostic laboratories for mutation detection in tumour samples as well as in inherited diseases. However, single biomarker assays will still remain important as confirmative assays, and they will also be used for cito diagnostics and in low-quality tissues. Comparing two DNA-based mutation detection methods with immunohistochemistry, our data show differences between these three methods with respect to the detection of different *BRAF* mutations. Sanger sequencing is specific and of course able to distinguish between V600E and V600K mutations. This well-established DNA-based method also enabled us to detect other alterations still of unknown importance. Compared to the LightMix assay, however, Sanger sequencing showed an inferior detection limit, indicating that higher tumour cell content in the sample is required to detect any genetic alterations. In the mCRC samples, we identified two additional V600-mutated tumours by applying the melting point assay. In the melanoma samples, we detected 15 additional mutations. Eight of these tumours were from patient with other mutation-positive tumours, but importantly, seven of these tumours were from six different patients with tumours classified by Sanger sequencing as wild type, and, thus, they would potentially have been excluded from treatment with *BRAF* inhibitors when relying on Sanger sequencing alone. A likely explanation for this could be that only a small fraction of the tumour cells in a sample harboured the mutation. Hence, a more sensitive assay with clamped wild-type amplification, such as the LightMix-assay, detects more mutations. For the moment, *BRAF* inhibitors are limited to treatment of metastatic melanomas only. For such patients, the clinical benefit of diagnosing a *BRAF* mutation in a very small fraction of the tumour cells may be questioned. However, assuming clonal expansion of *BRAF*-mutated cells due to their high growth rate, a negative result by Sanger sequencing may preclude effective treatment at a later stage, provided repeated sampling and testing are not performed. Considering the potential for anti-*BRAF* therapy in the adjuvant setting, a highly sensitive assay detecting minor subclones of *BRAF*-mutated cells, able to set out metastases, could be highly important. Another possible explanation is that all tumour cells in a mutated sample harbour the mutation, but that the percentage of tumour cells in the sample is too low for Sanger sequencing to detect the mutation. This is supported by the fact that for the IHC positive samples, the staining was homogenous with equal intensity throughout the majority of tumour cells.

Mutation-specific immunohistochemistry for detection of mutated *BRAF* protein introduced by Capper et al. in 2011 presented a method without the need for DNA isolation. Several publications [9, 13–16] have pointed out VE1 antibody’s excellent sensitivity and specificity, and some investigators [8, 23] conclude that IHC might be used as a screening for the mutation due to a 100 % specificity and that the uncertain or negative samples are left to be analysed by DNA-based methods. In line with these previous findings, we observed high concordance between DNA-based methods and IHC analysis in our melanoma samples. For all discrepancies, IHC was negative while DNA analysis detected the mutation. Thus, for melanoma, we find that IHC screening of samples might be considered but that samples testing negative for V600E should be subjects to DNA-based methods.

Importantly, we also assessed the performance of IHC in colorectal cancer specimens. In our mCRC dataset, we see a large discrepancy between the DNA-based methods and the IHC data. Based on the IHC data, we would miss two mCRC patients with the mutation, as well as suspect the mutation in one or more tumours from ten patients found to be wild type by the DNA-based assays. We do not have a full explanation to this finding which presents as unspecific staining in the colorectal cancer metastasis. One may speculate that this staining could be due to cross reactions in samples with particularly high levels of wild-type *BRAF* protein or toward other proteins; at this stage, however, no conclusion can be drawn. Nevertheless, unspecific staining does seem to occur, a worrisome situation with respect to the use of the IHC method. Especially since several diagnostic pathology laboratories tend to choose IHC as a fast pre-screening tool. IHC also had more uninterpretable cases, as compared to the DNA-based methods which had very low failure rates. Notably, our findings are in line with the results reported by Adackapara et al. in 2013 [17], strongly indicating that although the VE1 antibody may be useful for melanoma samples, its value for analysing colorectal cancer specimens is limited.

One could argue that our cutoffs for IHC are inaccurate, but if only samples scored as 3+ with respect to staining were to be considered positive, we would miss yet another mutated sample and we would still have two patients suspected to have mutated tumours despite negative DNA analysis. On the other hand, if we were to include the 1+ samples as positive, like Capper et al. did in their first paper [9], we would increase the number of suspected mutations to 101/285 which would clearly be wrong based on the data from the DNA-based methods as well as the previously reported frequencies of *BRAF* mutations in primary CRC (10 %) as well as in surgically removed metastasis (∼2 %) [31, 32]. Based on the DNA-based methods, we detected a mutation frequency of 5 % in our material which is slightly higher than that expected in surgically removed liver metastases. *BRAF*-mutated colorectal cancers have an unfavourable prognosis, and, thus, these patients are less likely to experience liver-limited disease and the option of surgical treatment of their metastases. For melanoma samples, we found a relatively low mutation frequency based on Sanger sequencing (40 %), but using the more sensitive LightMix assay, we reached a frequency of 48 %, closer to what is reported in the literature (approximately 60 %) [28].

All samples were evaluated for tumour content by an experienced pathologist, and it varied from 10 to 100 % (for the CRC samples, only five harboured <30 % tumour cells, for the MM samples, 7). For the discrepant results, low tumour cell content might explain the cases where the sample is classified as wild type by Sanger sequencing only and as mutated by IHC and LightMix. However, it cannot explain the cases where IHC is negative while both the DNA-based methods detect the mutation, and even more importantly, it cannot explain the false positive results in the colorectal cancer samples.

## Conclusions

Taken together, our data indicate that for high sensitivity, specific detection of *BRAF* V600E or V600K mutation, the LightMix high-resolution melting assay would be preferable. In order to be able to detect other mutations than these two specifically, Sanger sequencing would still be the method of choice despite its inferior sensitivity. IHC may be useful as a screening tool guiding further analytical approaches for malignant melanomas, but for colorectal cancer samples, IHC with the current antibody would not be recommendable for clinical tests.

## Electronic supplementary material

Below is the link to the electronic supplementary material.

ESM 1 (DOCX 44 kb)
