# Supplementary material for: Performance comparison of three BRAF V600E detection methods in malignant melanoma and colorectal cancer specimens
Source: Tumour Biol. 2014 Oct 16;36(2):1003–13. doi: 10.1007/s13277-014-2711-5 (PMC4342512; doi:10.1007/s13277-014-2711-5)
Supplement: Supplementary file 1 — (DOCX 44 kb) [file 13277_2014_2711_MOESM1_ESM.docx]

**Online supplementary information**

# Performance comparison of three *BRAF* V600E detection methods in malignant melanoma and colorectal cancer specimens

Inger Marie Løes, Heike Immervoll, Jon-Helge Angelsen, Arild Horn, Jürgen Geisler, Christian Busch, Per Eystein Lønning, Stian Knappskog

Inger Marie Løes, Christian Busch, Per Eystein Lønning, Stian Knappskog

Department of Clinical Science, University of Bergen, Bergen, Norway

Inger Marie Løes, Jürgen Geisler, Per Eystein Lønning, Stian Knappskog

Department of Oncology, Haukeland University Hospital, Bergen, Norway

Heike Immervoll

Department of Pathology, Ålesund Hospital, Ålesund, Norway

Jon-Helge Angelsen

Department of Clinical Medicine, University of Bergen, Bergen Norway

Jon-Helge Angelsen, Arild Horn

Department of Digestive Surgery, Haukeland University Hospital, Bergen, Norway

Jürgen Geisler

Current addresses: Institute of Clinical Medicine, University of Oslo, Norway and Department of Oncology, Akershus University Hospital, Norway

Christian Busch

Current address: Aleris Helse, Marken 34, 5017 Bergen, Norway

***Corresponding author:**

Stian Knappskog, Senior Scientist,

Department of Clinical Science,

University of Bergen,

Post-box 7804,

N-5021 Bergen

Norway

E-mail: [Stian.Knappskog@k2.uib.no](mailto:Stian.Knappskog@k2.uib.no)

Phone (+47) 55976447

Fax (+47) 55972046

| Sample ID | **Sanger** | **LightMix** | **IHC** | **% tumour** |
| --- | --- | --- | --- | --- |
| MM01-2 | wt | wt | wt | **20** |
| MM01-4 | wt | wt | wt (1+) | **50** |
| MM01-5 | wt | wt | wt | **60** |
| MM01-6 | wt | wt | wt | **80** |
| MM06-1 | wt | wt | *NA* | **NA** |
| MM09-2 | **V600E** | **V600E** | *NA* | **0** |
| MM10-1 | wt | **V600E** | **V600E (3+)** | **50** |
| MM10-2 | **V600E** | **V600E** | **V600E (3+)** | **40** |
| MM11-1 | wt | wt | *NA* | **0** |
| MM11-2 | **V600E** | **V600E** | **V600E (2+)** | **80** |
| MM11-3 | **V600E** | **V600E** | **V600E (3+)** | **60** |
| MM11-4 | wt | **V600E** | *NA* | **0** |
| MM11-5 | **V600E** | **V600E** | **V600E (2-3+)** | **70** |
| MM11-6 | **V600E** | **V600E** | **V600E (3+)** | **90** |
| MM12-1 | **V600K** | **V600K** | wt | **80** |
| MM12-2 | **V600K** | **V600K** | wt | **90** |
| MM14-2 | wt | wt | *NA* | **0** |
| MM16-1 | **V600E** | **V600E** | **V600E (3+)** | **70** |
| MM16-2 | **V600E** | **V600E** | **V600E (3+)** | **90** |
| MM18-1 | wt | wt | *NA* | **70** |
| MM19-1 | **V600E** | **V600E** | **V600E (3+)** | **90** |
| MM19-3 | **V600E** | **V600E** | **V600E (3+)** | **90** |
| MM19-4 | **V600E** | **V600E** | **V600E (3+)** | **40** |
| MM20-2 | wt | wt | *NA* | **0** |
| MM22-1 | **V600E** | **V600E** | **V600E (3+)** | **50** |
| MM22-2 | **V600E** | **V600E** | *NA* | **0** |
| MM22-3 | **V600E** | **V600E** | **V600E (3+)** | **80** |
| MM24-1 | **V600E** | **V600E** | **V600E (2-3+)** | **90** |
| MM24-2 | **V600E** | **V600E** | **V600E (2+)** | **<10** |
| MM25-1 | **V600E** | **V600E** | **V600E (3+)** | **70** |
| MM25-2 | **V600E** | **V600E** | **V600E (3+)** | **NA** |
| MM25-3 | **V600E** | **V600E** | wt | **60** |
| MM30-1 | **V600E** | **V600E** | **V600E (3+)** | **40** |
| MM31-1 | wt | wt | wt (1+) | **100** |
| MM31-2 | wt | wt | wt | **90** |
| MM32-1 | wt | wt | wt (1+) | **90** |
| MM32-2 | wt | wt | wt | **100** |
| MM34-1 | wt | wt | wt (0-1) | **100** |
| MM34-2 | wt | wt | wt | **70** |
| MM38-1 | **V600E** | **V600E** | **V600E (3+)** | **70** |
| MM38-3 | **wt** | **V600E** | *NA* | **NA** |
| MM40-2 | **V600E** | **V600E** | **V600E (2+)** | **30** |
| MM40-3 | **V600E** | **V600E** | **V600E (3+)** | **30** |
| MM42-1 | wt | wt | wt(0-1) | **40** |
| MM43-1 | **V600E** | **V600E** | **V600E (3+)** | **90** |
| MM43-2 | **V600E** | **V600E** | *NA* | **NA** |
| MM44-1 | **V600E** | **V600E** | **V600E (2-3+)** | **10** |
| MM44-2 | **V600E** | **V600E** | **V600E (3+)** | **20** |
| MM45-1 | **V600E** | **V600E** | **V600E (2-3+)** | **70** |
| MM50-1 | wt | wt | wt | **50** |
| MM51-1 | wt | wt | wt(0-1) | **90** |
| MM52-1 | **V600K** | **V600K** | wt | **100** |
| MM52-2 | **V600K** | **V600K** | wt | **90** |
| MM53-1 | **V600E** | **V600E** | **V600E (2-3+)** | **40** |
| MM53-2 | **V600E** | **V600E** | **V600E (2-3+)** | **80** |
| MM55-1 | **V600E** | **V600E** | **V600E (3+)** | **40** |
| MM58-1 | **V600E** | **V600E** | **V600E (2+)** | **70** |
| MM58-2 | **V600E** | **V600E** | **V600E (2+)** | **<10** |
| MM60-1 | **V600E** | **V600E** | **V600E (2+)** | **60** |
| MM61-1 | **V600E** | **V600E** | **V600E (3+)** | **70** |
| MM61-2 | wt | **V600E** | **V600E (2-3+)** | **20** |
| MM61-3 | **V600E** | **V600E** | wt (1+) | **80** |
| MM61-4 | **V600E** | **V600E** | wt | **70** |
| MM61-5 | **V600E** | **V600E** | *NA* | **0** |
| MM66-2 | wt | wt | wt | **90** |
| MM68-1 | wt | wt | wt | **90** |
| MM71-2 | wt | **V600E** | wt | **<10** |
| MM78-1 | wt | **V600E** | wt (1+) | **30** |
| MM82-1 | **V600E** | *NA* | *NA* | **70** |
| MM83-1 | **V600E** | **V600E** | **V600E (3+)** | **90** |
| MM83-2 | wt | **V600E** | *NA* | **1** |
| MM85-2 | **V600E** | **V600E** | **V600E (2+)** | **80** |
| MM86-1 | **V600E** | **V600E** | **V600E (2-3+)** | **80** |
| MM88-1 | wt | wt | wt (1+) | **80** |
| MM93-1 | **V600E** | **V600E** | **V600E (2+)** | **70** |
| MM94-1 | **V600E** | **V600E** | **V600E (3+)** | **100** |
| MM95-1 | **V600E** | **V600E** | **V600E (3+)** | **80** |

**Colorectal cancer samples**

| **Sample ID** | **Sanger** | **LightMix** | **IHC** | **% tumour** |
| --- | --- | --- | --- | --- |
| t1-1 | **V600E** | **V600E** | **V600E (2+)** | **20** |
| t1-2 | **V600E** | **V600E** | **V600E (3+)** | **100** |
| t7-1 | wt | wt | wt (1+) | **85** |
| t7-4 | wt | wt | wt (1+) | **70** |
| t9-1 | wt | wt | **V600E (2+)** | **100** |
| t9-2 | wt | wt | wt (1+) | **100** |
| t9-3 | wt | wt | **V600E (2+)** | **100** |
| t11-1 | wt | wt | wt | **90** |
| t11-2 | wt | wt | wt (1+) | **90** |
| t14-1 | wt | wt | **V600E (2+)** | **100** |
| t14-2 | wt | wt | wt | **50** |
| t15-1 | wt | **V600E** | **V600E (3+)** | **80** |
| t15-2 | **V600E** | **V600E** | **V600E (3+)** | **70** |
| t15-3 | **V600E** | **V600E** | **V600E (3+)** | **90** |
| t19-1 | wt | wt | wt (1+) | **100** |
| t19-2 | wt | wt | *NA* | **80** |
| t25-1 | wt | wt | wt | **100** |
| t25-2 | wt | wt | *NA* | **100** |
| t25-4 | wt | wt | *NA* | **100** |
| t25-5 | wt | wt | wt | **100** |
| t26-1 | wt | wt | wt (1+) | **80** |
| t26-2 | wt | wt | wt (1+) | **50** |
| t26-3 | wt | wt | wt | **100** |
| t26-4 | wt | wt | wt (1+) | **80** |
| t26-5 | wt | wt | **V600E (3+)** | **80** |
| t29-1 | wt | wt | wt | **100** |
| t29-2 | wt | wt | wt | **80** |
| t29-3 | wt | wt | wt | **30** |
| t29-4 | wt | wt | **V600E (2+)** | **100** |
| t30-1 | wt | wt | *NA* | **10** |
| t30-2 | wt | wt | **V600E (2+)** | **20** |
| t39-1 | **V600E** | **V600E** | wt (1+) | **100** |
| t39-2 | **V600E** | **V600E** | wt | **90** |
| t39-3 | **V600E** | **V600E** | wt | **90** |
| t42-1 | wt | wt | wt | **90** |
| t42-2 | wt | wt | wt | **10** |
| t42-3 | wt | wt | *NA* | **100** |
| t49-2 | wt | wt | **V600E (2+)** | **40** |
| t58-1 | wt | wt | wt | **80** |
| t58-2 | wt | wt | wt | **90** |
| t58-3 | wt | wt | wt (1+) | **90** |
| t58-4 | wt | wt | wt | **80** |
| t58-6 | wt | wt | wt | **100** |
| t58-7 | wt | wt | wt | **70** |
| t58-8 | wt | wt | **V600E (2+)** | **50** |
| t64-1 | wt | wt | wt | **100** |
| t64-2 | wt | wt | wt | **100** |
| t64-3 | wt | wt | **V600E (2+)** | **80** |
| t64-4 | wt | wt | **V600E (2+)** | **80** |
| t64-5 | wt | wt | wt | **90** |
| t64B-1 | wt | wt | wt | **70** |
| t64B-3 | wt | wt | wt | **80** |
| t64B-4 | wt | wt | wt | **100** |
| t67-1 | wt | wt | wt | **100** |
| t67-2 | wt | wt | **V600E (2+)** | **20** |
| t70-1 | wt | wt | wt | **50** |
| t70-2 | wt | wt | wt | **90** |
| t73-1 | wt | wt | wt | **80** |
| t73-2 | wt | wt | wt | **60** |
| t73-3 | wt | wt | wt | **90** |
| t73-4 | wt | wt | wt | **100** |
| t73-6 | wt | wt | *NA* | **100** |
| t75-1 | **V600E** | **V600E** | wt | **100** |
| t75-3 | **V600E** | **V600E** | wt | **100** |
| t81-1 | wt | wt | wt | **100** |
| t81-2 | wt | wt | wt | **100** |
| t82-1 | wt | wt | wt | **100** |
| t82-2 | wt | wt | wt | **100** |
| t84-1 | **V600E** | **V600E** | **V600E(2-3+)** | **100** |
| t84-2 | **V600E** | **V600E** | **V600E (3+)** | **100** |
| t84-3 | **V600E** | **V600E** | **V600E (2+)** | **80** |
| t84B-1 | **V600E** | **V600E** | wt | **100** |
| t85-1 | wt | wt | wt | **60** |
| t85-2 | wt | wt | *NA* | **70** |
| t85-3 | wt | wt | wt | **80** |
| t85-4 | wt | wt | wt | **40** |
| t85-5 | wt | wt | *NA* | **70** |
| t85-7 | wt | wt | wt | **50** |
| t93-1 | wt | wt | wt | **80** |
| t93-2 | wt | wt | wt | **100** |
| t93-3 | wt | wt | wt | **90** |
| t95-1 | **G/A 1780** | wt | wt (0-1+) | **70** |
| t97-1 | wt | wt | wt | **90** |
| t97-2 | wt | wt | wt | **80** |
| t98-1 | wt | wt | wt | **70** |
| t98-2 | wt | wt | wt | **90** |
| t99-1 | **V600E** | **V600E** | **V600E (2+)** | **95** |
| t99-2 | **V600E** | **V600E** | wt | **100** |
| t99-3 | **V600E** | **V600E** | wt (0-1+) | **90** |
| t99-4 | **V600E** | **V600E** | wt (1+) | **60** |
| t105-1 | wt | wt | *NA* | **80** |
| t105-2 | wt | wt | *NA* | **80** |
| t108-1 | wt | wt | wt | **30** |
| t108-2 | wt | wt | wt | **100** |
| t108-3 | wt | wt | wt | **100** |
| t110-1 | wt | wt | wt | **20** |
| t113-1 | wt | wt | wt | **70** |
| t126-2 | **ins TAC fom1797** | ^a^ | wt | **100** |
| t129-1 | wt | wt | wt | **80** |
| t132-1 | wt | wt | wt | **90** |
| t132-2 | wt | wt | wt | **95** |
| t138-1 | **V600E** | **V600E** | **V600E (3+)** | **100** |
| t138-2 | **V600E** | **V600E** | **V600E (3+)** | **80** |
| t138-3 | **V600E** | **V600E** | **V600E (3+)** | **100** |
| t138-4 | **V600E** | **V600E** | **V600E (3+)** | **100** |
| t145-1 | wt | wt | wt (1+) | **100** |
| t149-3 | wt | wt | wt (1+) | **80** |
| t156-1 | wt | wt | **V600E (2+)** | **100** |
| t156-2 | wt | wt | wt (0-1+) | 70 |

^a^ shift in melting curve different than V600E and V600K

**Suppl. Methods: A presentation of details on the establishment of IHC methods for the mutation specific antibody VE1.**

We tested several positive and negative controls; negative controls were normal tissue (intestinal mucosa and skin) as well as different tumours which had tested negative by sequencing.

Positive controls were several tumours found to be *BRAF* V600E positive by sequencing.

We chose the protocol for Ventana resulting in the highest signal to noise ratio. The procedure is presented in the material and methods section.

During the establishment of the protocol we also tried the CC2 (buffer with low pH) on the Ventana platform, but that did not work well.

Several different time periods for incubation- and epitope retrieval were tried as well as different dilutions of the primary antibody.

For comparison with Ventana, we performed manual immunohistochemistry, and we also analysed the samples on the DAKO autostainer.

The control samples were analysed without pre-treatment as well as after enzyme treatment (Histo/Zyme) and proteinase K.

We tested several different buffers including citrate buffer (pH 6), citraconic anhydride (pH 6, 1), Tris buffer (pH 10), Tris EDTA (pH 9) and TRS buffer (DAKO) (pH 6, 1).

For citraconic anhydride we tried low temperature antigen retrieval (LTAR)

For citrate buffer LTAR and high temperature antigen retrieval (HTAR)

Tris buffer: LTAR and HTAR

Tris EDTA: LTAR and HTAR.

TRS buffer: LTAR

Several dilutions of the primary antibodies and 2 different antibody diluents with different sodium content were tried out in combination with the different detection kits/systems MACH3 from Biocare and Envision Flex (Dako).

Envision FLEX (Dako) and MACH3 (Biocare) with Tris EDTA-HTAR, produced acceptable results for VE1, but OptiView Universal DAB Detection Kit (Ventana) using CC1 (buffer with high pH) gave better signal to noise ratio and proved to be better "over all".

All incubations with the antibodies for detection by MACH3 (Biocare) and Envision Flex (Dako) were performed at room temperature. We did not attempt to incubate the primary antibody VE1 at 37 degrees using MACH3 and Envision Flex.

The reason why the results came out best using the Ventana platform is most likely due to incubation by the antibody at 37 degrees which seems to be an advantage for some antibodies compared to incubation at room temperature or 4 degrees used in some cases.
